# A long noncoding RNA promotes cellulase expression in *Trichoderma reesei*

**DOI:** 10.1186/s13068-018-1081-4

**Published:** 2018-03-23

**Authors:** Petra Till, Marion E. Pucher, Robert L. Mach, Astrid R. Mach-Aigner

**Affiliations:** 1 Christian Doppler Laboratory for Optimized Expression of Carbohydrate-active Enzymes, Institute of Chemical, Environmental and Bioscience Engineering, TU Wien, Gumpendorfer Str. 1a, 1060 Vienna, Austria; 2 Institute of Chemical, Environmental and Bioscience Engineering, TU Wien, Gumpendorfer Str. 1a, 1060 Vienna, Austria

**Keywords:** *Trichoderma reesei*, *Hypocrea jecorina*, Filamentous fungi, Long noncoding RNA, *HAX1*, Cellulases

## Abstract

**Background:**

Due to its capability to secrete large quantities of plant biomass degrading enzymes (PBDE), *Trichoderma reesei* is widely applied for industrial purposes. In nature, expression of PBDE is efficiently regulated in this fungus. Several factors involved in this regulatory network have been identified. However, most of them are transcription factors. Long noncoding RNAs (lncRNAs) emerged as common players acting on epigenetic or transcriptional regulation in several eukaryotic organisms. To date, no lncRNA has been described in filamentous fungi.

**Results:**

A lncRNA termed *HAX1* was identified in *T. reesei* QM9414. In this study, it was characterized and evidence for its regulatory impact on cellulase expression was provided. Interestingly, different versions of *HAX1* were identified in different strains (namely, QM6a, QM9414, and Rut-C30), varying in terms of RNA length. Remarkably, considerable longer variants of this lncRNA are present in hypercellulolytic strains compared to the wild-type strain QM6a. Based on these results, a correlation between RNA length and the functional impact of *HAX1* on PBDE expression was supposed. This assumption was verified by overexpressing the most abundant *HAX1* versions identified in QM6a, QM9414, and Rut-C30. Such *HAX1* overexpression on the one hand was suitable for regaining the function in *hax1* disruption strains, and on the other hand resulted in notably higher cellulase activities in QM6a, especially by the expression of longer *HAX1* versions.

**Conclusion:**

With *HAX1,* for the first time the regulatory role of a lncRNA in filamentous fungi was uncovered. Besides this, a new player involved in the complex regulation of PBDE expression in *T. reesei* was identified. Due to its enhancing effect on cellulase activity, *HAX1* was shown to be not only interesting for basic research, but also a promising candidate for expanding the set of biotechnological tools for industrial application of *T. reesei*.

**Electronic supplementary material:**

The online version of this article (10.1186/s13068-018-1081-4) contains supplementary material, which is available to authorized users.

## Background

The filamentous fungus *Trichoderma reesei* (teleomorph *Hypocrea jecorina* [1], phylum *Ascomycota*) is one of the most potent producers of plant biomass degrading enzymes (PBDE) used in industrial applications. Due to its saprophytic life style, it secretes large quantities of cellulases, hemicellulases, and other glycoside hydrolases such as β-glucosidases and xylosidases [2]. The dominating ones are the major cellobiohydrolases CBHI and CBHII (EC.3.2.1.91) [3] as well as the main *endo*-β-1,4-xylanases, XYNI and XYNII (EC.3.2.1.8) [4]. Industrial demand for these enzymes is given in food, feed [5], and textile [6] industries, paper and pulp fabrication [7], as well as production of biofuels [8–10]. In nature, production of cellulases and xylanases is tightly regulated in this fungus. Thus, understanding the processes involved in the regulatory network with the purpose of strain improvement are in the focus of past and present scientific research [11–15].

The wild-type strain QM6a is a good producer of PBDE under inducing conditions [16]. Yet, when more easily utilizable carbon sources, such as glucose, are available, the production of cellulases and xylanases is turned down by carbon catabolite repression (CCR) [17, 18]. Two strains derived from QM6a via random mutagenesis and selection for increased PBDE expression are QM9414 and Rut-C30 [19]. Rut-C30 is characterized by strongly increased cellulase activities and partial carbon catabolite derepression and thus became the progenitor of most industrial strains [19–21].

The reason for the partial carbon catabolite derepressed phenotype of Rut-C30 is the production of a truncated version of the main regulatory factor acting on CCR, the Carbon Catabolite Repressor 1 (Cre1) [22]. Besides Cre1, the main regulatory protein of PBDE expression in all *T. reesei* strains is the essential transactivator Xylanase regulator 1 (Xyr1). A deletion of this transactivator results in a complete shutdown of all major cellulases and hemicellulases [23, 24]. Other proteins functioning as regulators of PBDE expression, are Ace1 [25], Ace2 [26], Ace3 [27], Cre2 [28], PAC1 [29], and the recently reported BglR [30]. However, all of them are modulating the expression of their target genes by operating as transcription factors.

Another widespread mechanism for specific regulation of gene expression in eukaryotes that has gained in importance more and more in the past decades is accomplished by noncoding RNAs (ncRNAs). A large group of functional ncRNAs are the long noncoding RNAs (lncRNAs). They are mainly defined upon their size of > 200 nt and by their functional, but non-protein coding nature [31–35]. As lncRNAs identified in different organisms obviously do not share a common evolutionary origin, their functional roles, mechanistic strategies, and features are very diverse [36]. They might possess a poly(A)-tail and could get processed by splicing, but both is not necessarily the case. However, as they act directly as RNAs in a functional way, they are usually characterized by distinct secondary structure formation [37, 38]. Given a regulatory role, the expression of the lncRNAs is often tightly regulated either in a tissue- or in a condition-specific manner, and generally low compared to mRNAs [31, 34, 39]. LncRNAs are present in several eukaryotes such as plants [40], invertebrates [41, 42], or amphibians [43]. Yet, the most prominent examples of lncRNAs have been described in mammals and are predominantly affecting epigenetic regulation. Among those are *HOTAIR*, a transregulator of the *Hox* cluster that is widely involved in gene silencing [44]; and the central factor for X-chromosomal inactivation, *Xist* [45]. In addition, in yeast, lncRNAs causing transcriptional gene silencing by acting on the chromatin status and epigenetic marks have been identified [36]. Two more accurately described lncRNAs in yeast are *SRG1* [46] and an *fbp1*-proximal element involved in glucose starvation [47], both of them functioning in *cis* in a transcription-interfering manner. Compared to mammalian lncRNAs, examples in yeast are rare and usually neither named nor investigated in detail. Considering the impressively high level of nc transcripts compared to protein encoding genes [34, 35, 48, 49] and the increasing number of lncRNAs found throughout the whole eukaryotic domain, lncRNAs are likely to play a role in all eukaryotic organisms. However, to our knowledge, hitherto no lncRNAs have been identified in filamentous fungi.

During this study, a lncRNA in the industrially applied fungus *T. reesei* was discovered. Evidence for its impact on regulation of PBDE expression was provided. Variation of this lncRNA in length in moderate cellulase producing compared to overproducing *T. reesei* strains was shown, hence indicating evolutionary improvement of the lncRNA during strain genesis. As a final outcome of this study, this lncRNA is not only the first description of such in filamentous fungi, but also highlighted as an interesting player in a complex regulatory network and as a promising biotechnological tool for strain improvement in *T. reesei*.

## Results

### An undescribed gene locus influences cellulase expression in *T. reesei* QM9414

**Fig. 1:**
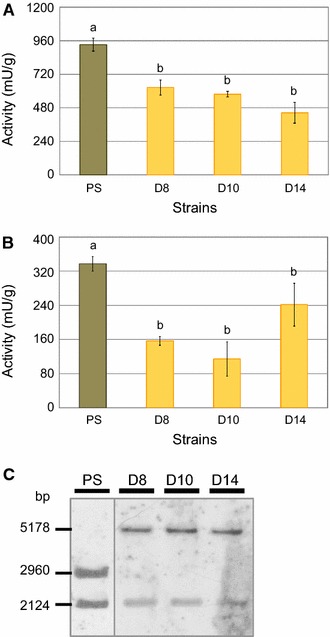
Phenotype and genotype of *Trichoderma reesei* QM9414 disruption mutants. The β-glucosidase activities** A** and cellulase activities **B** of three *T. reesei* mutant strains bearing a randomly integrated *amdS* marker gene (i.e., QM9414_D*hax1*_8 (D8), _10 (D10) and _14 (D14); yellow bars) and their parent strain QM9414 (PS; brown bars). Strains were cultivated on xylan for 66 h (for β–glucosidase assays) and on lactose for 48 h (for cellulase assays). Reaction with the Azo-cellazyme C tablet for detection of cellulase activity was performed for 90 min. Enzymatic activities are given in μ and referred to biomass (dry weight). The values are means of biological triplicates. The error bars depict the standard deviation and different letters denote statistical difference among compared data employing ANOVA (*P* < 0.05). **C** Southern blot analysis using *Sac*II-digested chromosomal DNA of the investigated strains and a locus-specific probe. Expected signals are at 5178 bp (from integration of the *amdS* cassette at this locus), 2960 bp (for the native locus) and 2124 bp (for a sequence downstream of the *amdS* targeted locus)

Commonly, phenotypic investigation of strains generated by targeted mutagenesis requires a reference strain carrying solely the marker gene. For this reason, the *amdS* marker gene enabling utilization of acetamide [50] was randomly integrated into the genome of *T. reesei* QM9414 (ATCC 26921). To our surprise, subsequent characterization studies of the 18 resulting reference strains revealed certain phenotypic characteristics of three candidates. Enzymatic assays of liquid fungal cultures grown on xylan showed that β-glucosidase activity is significantly lower in these strains compared to the parent strain QM9414, i.e, about 59% (Fig. 1A). Moreover, also the cellulase activity was reduced about 50% in cultures that were induced on lactose (Fig. 1B).

Since enzymes acting on the degradation of cellulose were affected in these strains, a crucial role of the disrupted genomic region in regulation of cellulose degradation was hypothesized. Hence, the site targeted by the *amdS* marker gene was identified via inverse PCR. Interestingly, sequence analysis revealed that—although a random integration strategy was followed—the *amdS* gene was integrated at the same locus in all three strains. This finding was verified by Southern blot analysis applying a locus-specific probe (Fig. 1C). The reason for the preference of this target site remained unclear; however, the experiment resulted in the identification of a hitherto undescribed region of the genome probably involved in regulation of PBDE expression.

### Discovery of *HAX1*, a long noncoding RNA

**Fig. 2:**
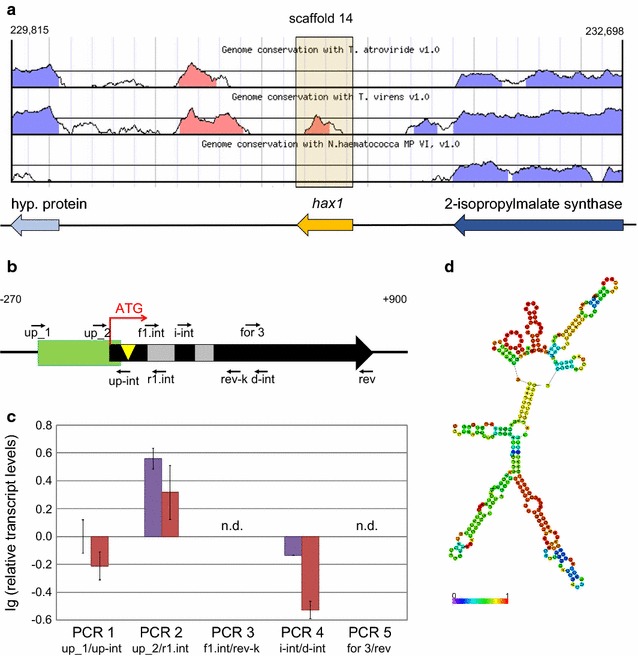
Properties of the *hax1* locus and its encoded regulatory factor. **a** Locus targeted by *amdS* integration in QM9414_D*hax1* strains and its genetic environment according to JGI genome database of *Trichoderma reesei* QM6a v2.0. The targeted locus *hax1*, the downstream coding region referred to as “hypothetical protein” (hyp. protein) and the upstream gene encoding 2-isopropylmalate synthase are illustrated as yellow, light and dark blue arrows, respectively. **b** Schematic illustration of the 782 bp *hax1* structural gene (black arrow) predicted by the web server AUGUSTUS. Start codon (ATG, red arrow), introns (grey boxes), site targeted by integration of the *amdS* cassette (yellow triangle), 5′-region rich in Xyr1-binding sites (green box) and position of the primers used for transcript analysis (thin, black arrows) are indicated. Numbers on top indicate position from ATG in base pairs. **c** Transcript levels of *hax1* in QM9414 that was pre-grown and transferred to medium without carbon source (violet bars) or 1% (w/v) d-glucose (red bars) for 3 h. Indicated primer pairs were used for PCR 1, 2, 3, 4, and 5, respectively. Transcript levels were normalized to *act* and *sar1*, refer to PCR 1 (no carbon source) and are given in logarithmic scale (lg). Analysis was performed in technical triplicates. Error bars indicate standard deviations. Up_1, up-hax1 for_1; up-int, hax1 rev up-Intron; up_2, up-hax1 for_2; r1.int, hax1 rev 1.Intron; f1.int, hax1 for_1.Intron; rev-k, hax1 rev kurz; i-int, hax1 for_inter-Intron; d-int, hax1 rev_down-intron; for 3, hax1 for_3; rev, hax1 rev; n.d., not detectable. **d** In silico structure prediction of the lncRNA *HAX1* with minimized free energy. Structure stability is increasing from violet, blue, green and yellow to red

Based on the genome sequence of *T. reesei* QM6a accessible in the Joint Genome Institute (JGI) database [51], the site targeted by the *amdS* marker gene was assigned to an intergenic region on scaffold 14. It is flanked upstream by a 2-isopropylmalate synthase (Protein ID 79495), and downstream by a poorly described coding region referred to as “hypothetical protein” (Protein ID 108999); both of them are minus-strand encoded (Fig. 2a). Remarkably, the targeted region seems to lack any homologues. Genome conservation analysis displayed in Fig. 2a indicates only slight sequence similarities between *T. reesei* to a noncoding region on the *Trichoderma virens* genome and no homolog regions in *Nectria haematococca* or *Trichoderma atroviridae*.

To find out if the observed cellulase-depleted phenotype of the disruption strains is caused by the interruption of the undescribed genomic region or rather by any impact of the integrated *amdS* cassette on expression of the adjacent genes, transcript analysis of the genes encoding the hypothetical protein and the 2-isopropylmalate synthase was performed. For this purpose, the three mutants and their parent strain QM9414 were pre-grown and replaced to a cellulase inducing and a repressing condition and compared to a replacement to a medium without carbon source. As given in an additional file, transcript levels of both adjacent genes are the same in QM9414 and the mutant strains (Additional file 1). Thus, an effect of *amdS* integration on the cellulase activity by altering the expression of the upstream- or downstream genes can be excluded. In addition, a particular effect of the used marker system was ruled out by generation of other disruption strains bearing the *hph* gene (encoding hygromycin B resistance) instead of the *amdS* gene at the same locus.

As a consequence, the interrupted region itself was supposed to encode a gene involved in regulation of cellulose degrading enzymes. Hence, gene prediction was performed applying different gene prediction models for in silico analyses of the locus. One used algorithm provided by the gene prediction web server AUGUSTUS [52] assigned the sequence as coding for a gene. An illustration of the predicted, 782 bp-long structural gene is shown in Fig. 2b. It contains two putative introns at 113–201 and 269–325 bp from the ATG. At the 5′-end (untranslated- and coding region), a sequence element rich in Xyr1-binding sites is present.

Transcript formation of this potential gene further referred to as “*hax1*” was analysed via reverse transcription quantitative PCR (RT-qPCR) (Fig. 2c). To cover the whole structural gene, transcript levels of five overlapping fragments (each of them approximately 200–300 bp in length) were determined in separate PCRs. In general, transcript levels of *hax1* were quite low. However, transcript formation could be verified for the 5′-end of the gene (PCR 2) as well as for the 5′-upstream region (PCR 1). Lower levels could also be detected in case of the central region including the second intron (PCR 4), but no amplicons resulted for the central region comprising both introns (PCR 3) and for the 3′-end (PCR 5). This analysis suggested that not the whole predicted gene, but mainly, the 5′-terminal region is transcribed. Therefore, the generation of the potential translation product Hax1 is rather unlikely. This assumption is further supported by the detection of a transcript in the 5′-region that comprises at least a part of the putative first intron (Fig. 2c, PCR 2), suggesting that posttranscriptional splicing events do not take place. In addition, the analysis of the codon usage [53] of the protein encoding sequence displayed in an additional file, neither supports the presence of a typical *T. reesei* gene, nor does it indicate an origin in lateral gene transfer from another organism (Additional file 2). Finally, a protein BLAST of the deduced amino acid sequence as well as a nucleotide BLAST of the *hax1* gene to the pool of coding elements gathered in the NCBI archive [54] did not result in any hits.

Altogether, there are no indications for the generation of a translation product, even if transcript formation could be verified. These findings suggest that the region targeted by *amdS* insertion is a ncRNA. To concretely define the boundaries of this RNA, 3′ and 5′ rapid amplification of cDNA ends (RACE) were performed. 5′ RACE revealed a frequent occurrence of a 5′-end 127 nt upstream of the ATG. Classical 3′ RACE applying the supplied Oligo dT-Anchor primer for reverse transcription did not yield any specific products. Yet, enrichment of *HAX1* from QM9414 RNA extracts using a biotinylated *hax1*-specific DNA probe and streptavidin-linked magnetic beads followed by poly(A)-tailing and 3′ RACE defined the major 3′-end to be located 173 nt downstream of the ATG. This end could also be verified via *HAX1* enrichment and 3′ RACE, skipping the additional poly(A)-tailing step.

The resulting RNA is 299 nt in length, hence classifying it as a lncRNA. Since reverse transcription using the Oligo dT-Anchor primer for 3′ RACE analysis gave a specific product, *HAX1* is supposed to be 3′ polyadenylated. According to in silico structure prediction using the RNAfold Web server [55], it is characterized by a distinct secondary structure formation (Fig. 2d), which is a typical property of lncRNAs. To this end, the function of the lncRNA *HAX1* as a regulatory factor of PBDE expression was supposed.

### No phenotypic effects by *hax1*-disruption in the *T. reesei* wild-type strain QM6a

**Fig. 3:**
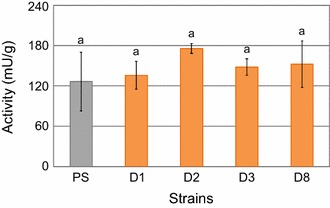
Cellulase activities of *Trichoderma reesei* QM6a_D*hax1* strains. QM6a_D*hax1* strains (D1, D2, D3, and D8; orange bars) and the parent strain QM6a_∆*tmus53* (PS; grey bar) were cultivated on lactose for 70 h. Reaction with the Azo-Cellazyme C tablet for detection of cellulase activity was performed for 30 min. Enzymatic activities measured in the resulting supernatants are given in μ and referred to the biomass (dry weight). The values are means of biological triplicates. The error bars depict the standard deviation and different letters denote statistical difference among compared data employing ANOVA (*P* < 0.05)

As the disruption of *hax1* led to a decreased cellulase expression in *T. reesei* QM9414, the same locus was disrupted in the wild-type strain QM6a by directed integration of the *amdS* gene. For this purpose, a disruption cassette comprising the 5′- and 3′-flanking regions of the targeted site for integration of the *amdS* marker gene was produced. Protoplast transformation led to integration of the cassette into the genome of QM6a_∆*tmus53* by homologous recombination. Four of the obtained disruption strains (QM6a_D*hax1*_1, _2, _3, and _8) were chosen for phenotypic analysis. Again, strains were grown in liquid media containing lactose for induction of cellulase expression. The detected enzymatic activities in the resulting culture supernatants are given in Fig. 3. Surprisingly, in *HAX1* disrupted strains, cellulase activity was not reduced compared to the parent strain. To learn if this apparent lack of the characteristic phenotype is caused by a failure in transcript formation, RT-qPCR was performed. Amplicons using primers for PCR 2 (compare Fig. 2c) appeared after 28.3 cycles for cultures grown without carbon source, and 27.3 cycles for cultivation on 1% d-glucose, yielding average relative logarithmic transcript levels of 0.00 and 0.26, respectively (see Additional file 3). These results demonstrate that, though transcript formation of *hax1* is given, the disruption of the locus had no effect on cellulase activity in case of the *T. reesei* wild-type strain QM6a. This raised the question if the role of the lncRNA *HAX1* might differ in the various strains of *T. reesei*.

### *HAX1*-length differs in different *T. reesei* strains

As the resulting phenotype from the disruption of *hax1* indicated differences in various *T. reesei* strains, 5′ and 3′ RACE for *HAX1* was performed also in QM6a and Rut-C30. In both strains, 3′ RACE of *HAX1* enriched from total RNA extracts verified the major 3′-end located 173 nt downstream of the ATG, as defined before in QM9414. In contrast, the 5′-ends of the lncRNA turned out to be different in the strains. Whereas in QM9414, the major transcription start point was located 127 nt upstream of the ATG, the most frequent 5′-end in QM6a was found 89/90 nt upstream of the ATG yielding a shorter transcript of 262 nt in length. Only rarely, longer versions of *HAX1* (5′-end up to − 151 nt) were detected in QM6a, just as in QM9414. However, the highest abundance of long *HAX1* versions was found in the hypercellulolytic strain Rut-C30. There, the major 5′-end is located − 255 nt of the ATG, albeit also in this case sporadically both longer (e.g., − 266 nt) and shorter (e.g., − 160 nt) versions were detected. This allows the possibility that not only the presence or the absence of *HAX1*, but also a particular length can have a distinct regulatory impact. To learn if the different levels of cellulase activities could result from variations in the genomic organisation of the *hax1* locus rather than from *HAX1* length, the locus was sequenced in QM9414 and Rut-C30 and aligned to the sequence of QM6a. Both the sequence of the locus and the position relative to the adjacent genes were the same in all three strains (see Additional file 4), hence pointing to the transcript length as the important difference. Provided that the RNA length is a relevant property, we investigated if it might be determined by a difference in RNA stability of the *HAX1* versions. We could not observe degradation of any of the three *HAX1* versions up to 2 h after the transcription was stopped by the transcription inhibitor DRB (see Additional file 5). Thus, we exclude that the different lengths are a result of different stability, but rather of a different transcription start site.

**Fig. 4:**
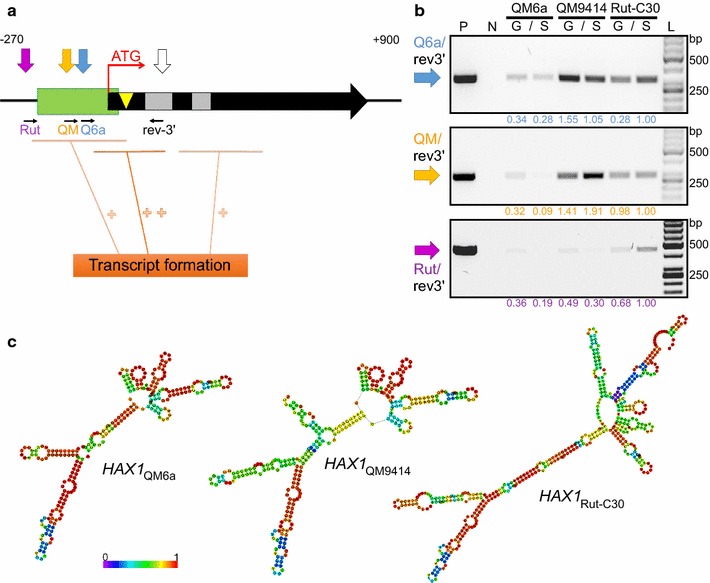
Analyses of *HAX1* versions in *T. reesei* QM6a, QM9414 and Rut-C30. **a** Schematic illustration of the 782 bp *hax1* structural gene (black arrow). Start codon (ATG, red arrow), introns (grey boxes), site targeted by integration of the *amdS* cassette (yellow triangle) and 5′-region rich in Xyr1-binding sites (green box) are indicated. Previously detected transcripts (Fig. 2c; PCR 1, PCR 2 and PCR 4) are illustrated by orange lines; lower or higher transcript levels are indicated by one or two plus symbols, respectively. Approximate positions of the 3′-end (white arrow) and 5′-ends of *HAX1*_QM6a_ (blue arrow), *HAX1*_QM9414_ (yellow arrow) and *HAX1*_Rut-C30_ (purple arrow) defined by RACE-PCR are marked. Positions of the primers used for amplification of the respective *hax1* versions displayed in **b** are given as thin, black arrows. Rev-3′, hax1 rev_3′QM6a; Q6a, hax1 for_QM6a_BcuI; QM, hax1 for_QM9414_BcuI; Rut, hax1 for_Rut-C30_BcuI. Numbers on top indicate position from ATG in base pairs. **b** Gel electrophoreses of PCRs of the *HAX1* versions in different *T. reesei* strains using cDNAs of QM6a, QM9414 and Rut-C30 as templates. Strains were pre-grown and transferred to medium with 1% (w/v) d-glucose (G) or 1.5 mM sophorose (S). Used primer pairs are indicated on the left (i.e, the PCR for detecting *hax1*_QM6a_ is analysed in gel on top; for detecting *hax1*_QM9414_ in the middle gel; and for detecting *hax1*_Rut-C30_ in the gel at the bottom). The strength of each band was quantified using Image Lab version 5.2 and related to the sample of Rut-C30 on sophorose (numbers are given below the respective band). P, Positive control; N, negative control; L, 50-bp DNA ladder. **c** In silico structure prediction of *HAX1*_QM6a_, *HAX1*_QM9414_ and *HAX1*_Rut-C30_ based on RACE-defined 3′- and 5′-ends. Structures of minimized free energy are displayed. The stability is increasing from violet, blue, green, and yellow to red

The respective ends of the major *HAX1* versions identified in QM6a (*hax1*_QM6a_), QM9414 (*hax1*_QM9414_), and Rut-C30 (*hax1*_Rut-C30_) are marked on the schematic illustration in Fig. 4a. Abundance of the transcript versions in the different strains was tested by a qualitative PCR using cDNAs as templates and a limited number of cycles to enable an estimation of the quantities. As expected, *hax1*_QM6a_ can be detected in all three strains, whereas the longer transcript versions *hax1*_QM9414_ and *hax1*_Rut-C30_ are mainly amplified from QM9414 and Rut-C30, respectively (Fig. 4b). Levels of *hax1*_Rut-C30_ were very low, giving only faint bands. Albeit this version is detectable in all three strains, it is predominantly detectable in samples derived from Rut-C30 compared to QM6a and QM9414, especially on sophorose as a cellulase inducing condition (Fig. 4b).

To estimate if *HAX1*_QM6a_, *HAX1*_QM9414,_ and *HAX1*_Rut-C30_ could fulfil different functions, in silico structure prediction was performed using the RNAfold Web server [55]. The secondary structures of minimized free energy are displayed in Fig. 4c. All of them are characterized by a lncRNA-typical formation of duplexed RNA. They are composed of a stem forming a hairpin-rich region at one end and splitting into two stem loops on the other end. In *HAX1*_QM6a_ and *HAX1*_QM9414,_ most of the hairpins are quite well conserved; however, in *HAX1*_QM9414,_ an additional hairpin is breaking the structure of the main stem. For *HAX1*_Rut-C30,_ in contrast, the overall structure is rather different. In fact, the three versions of *HAX1* only have one stem loop in common. This supports the assumption that those RNAs can act differently.

### Overexpression of *hax1* rescues the cellulase-depleted phenotype in QM9414_D*hax1* strains

**Fig. 5:**
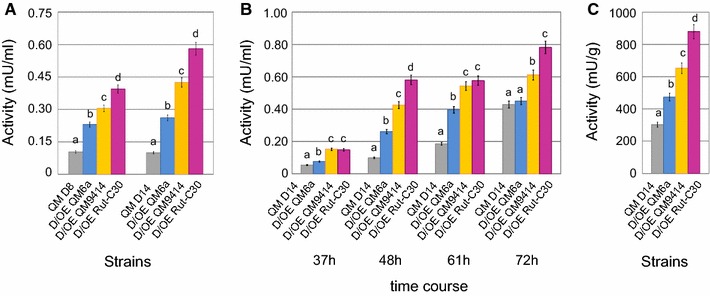
Cellulase activities of complemented QM9414_D*hax1* strains. Cultivation of QM9414_D*hax1* strains overexpressing either *hax1*_QM6a_ (D/OE QM6a; blue bars), *hax1*_QM9414_ (D/OE QM9414; yellow bars) or *hax1*_Rut-C30_ (D/OE Rut-C30; purple bars) and the respective parent strains QM9414_D*hax1*_8 and _14 (QM D8 and QM D14; grey bars) was performed on lactose for 72 h. **A** Cellulase activities of parent strains and the corresponding overexpression strains after cultivation on lactose for 48 h are given in reference to the culture volume. **B** Cellulase activities of the parent strain QM9414_D*hax1*_14 and the corresponding overexpression strains after cultivation on lactose for 37, 48, 61 and 72 h are given in reference to the culture volume. **c** Cellulase activities of the parent strain QM9414_D*hax1*_14 and the corresponding overexpression strains after cultivation on lactose for 72 h are given in reference to the final biomass (dry weight). Reaction with the Azo-Cellazyme C tablet for detection of cellulase activity was performed for 2 h, 45, 20, and 20 min for the timepoints 37, 48, 61, and 72 h, respectively. All values are means of biological triplicates. The error bars depict the standard deviation and different letters denote statistical difference among compared data employing ANOVA (*P* < 0.05)

To understand the distinct impact of *HAX1*_QM6a_, *HAX1*_QM9414,_ and *HAX1*_Rut-C30_, all three versions were overexpressed in two of the QM9414 *HAX1* mutant strains (i.e., QM9414_D*hax1_*8 and _14). As an initial control experiment, the absence of the three *hax1* versions was tested in the two recipient strains by RT-qPCR (see Additional file 6). The expression constructs comprise the native *bgl1* (β-glucosidase 1) promoter [56] and the respective version of *hax1,* which are flanked by the *pyr4* (encoding the orotidine 5′-phosphate decarboxylase) upstream- and downstream regions. Consequently, homologous integration at the *pyr4* locus resulted in a 5-fluoroorotic acid (5-FOA) resistant and uridine auxotroph phenotype. The usage of the *bgl1* promoter, of which the transcription start point is known, secured that only *hax1* is expressed. For characterization, the overexpression strains and the two parent strains were cultivated in liquid culture on 1% lactose for 72 h. Supernatant samples were taken after 37, 48, 60, and 72 h. Cellulase activity was increased in all overexpression strains compared to the parent strains after 48 h of cultivation (Fig. 5A). Moreover, differences resulting from overexpression of the distinct *hax1* version became evident: overexpression of *hax1*_QM6a_ has less impact on cellulase activity than *hax1*_QM9414_, and overexpression of *hax1*_Rut-C30_ yielded the highest cellulase activity. Even though the results slightly differed between the two strain lines, exactly the same trend could be found. As shown for QM9414_D*hax1*_14, this trend remains constant during the whole time course of the experiment (Fig. 5B) and can also be detected at the final time point if referred to the biomass (Fig. 5C).

### Overexpression of *hax1* enhances cellulase activity in *T. reesei* QM6a

**Fig. 6:**
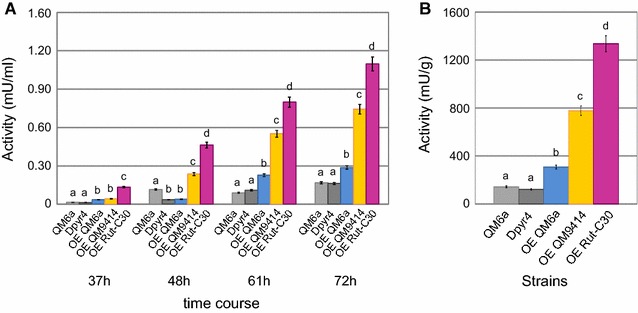
Cellulase activities of QM6a *hax1* overexpression strains. Cultivation of QM6a overexpressing either *hax1*_QM6a_ (OE QM6a; blue bars), *hax1*_QM9414_ (OE QM9414; yellow bars) or *hax1*_Rut-C30_ (OE Rut-C30; purple bars), the parent strain QM6a_∆*tmus53* (QM6a; light grey bars) and QM6a_∆*tmus53_*∆*pyr4* as a reference for a *pyr4* deleted background (Dpyr4; dark grey bars) was performed on lactose for 72 h. **A** Cellulase activities of the overexpression and reference strains after cultivation on lactose for 37, 48, 61 and 72 h are given in reference to the culture volume. **B** Cellulase activities of the overexpression and reference strains after cultivation on lactose for 72 h are given in reference to the final biomass (dry weight). Reaction with the Azo-Cellazyme C tablet for detection of cellulase activity was performed for 5, 3, 2 h and 75 min for the timepoints 37, 48, 61, and 72 h, respectively. All values are means of biological triplicates. The error bars depict the standard deviation and different letters denote statistical difference among compared data employing ANOVA (*P* < 0.05)

To learn if the expression of *hax1*_QM6a_, *hax1*_QM9414,_ and *hax1*_Rut-C30_ can change cellulase expression in the *T. reesei* wild-type strain QM6a, the before used constructs were introduced into QM6a_∆*tmus53* and integrated at the *pyr4* locus by homologous recombination. Subsequently, the resulting strains overexpressing either of the *hax1* versions as well as the parent strain (QM6a_∆*tmus53)* and a reference strain (QM6a_∆*tmus53*_∆*pyr4)* were characterized via enzymatic assays. Again, a time course experiment was performed growing the strains on 1% lactose for cellulase induction. The enzymatic activities of samples prepared after 37, 48, 60, and 72 h were determined. As observed before for *hax1* overexpression in QM9414_D*hax1*, the cellulase activities are importantly higher in all QM6a_OE*hax1* strains compared to the parent and reference strain (Fig. 6A). Again, depending on the expressed version of *HAX1*, different levels of cellulase activities were achieved: the overexpression of the longest *HAX1* version identified in Rut-C30 had the largest impact on cellulase activity. This is true for different times of cultivation as well as for values referred to the final biomass (Fig. 6A, B). Interestingly, in the case of the wild-type strain QM6a, the effect of overexpressing *hax1* was even more pronounced than in the case of QM9414. For example, the cellulase activities of strains overexpressing *hax1*_Rut-C30_ in QM9414_D*hax1* were threefold higher compared to the parent strain, whereas in QM6a_OE*hax1*_Rut-C30,_ they were even fivefold increased. Conclusively, the introduction of additional *hax1* copies (especially the long version *hax1*_Rut-C30_) did not only restore cellulase expression in *hax1*-disrupted strains, but it suggests itself as a tool for enhancing cellulase production.

## Discussion

In the last years, lncRNAs emerged as crucial factors acting on transcriptional and epigenetic regulation in eukaryotes. Within the fungal kingdom, the literature is only available about lncRNAs identified in yeast [36, 46, 47]. Thus, to our knowledge, the lncRNA *HAX1* characterized in this study is the first one described in a filamentous fungus. As a prime representative of this category, its features and mechanistic strategies are of special importance. In course of this work, *HAX1* was found to be 3′ polyadenylated, yet there is no information about the presence or absence of a 5′ methylguanosine cap. Anyhow, those are variable characteristic of lncRNAs [37, 38]. A more distinctive feature is the formation of pronounced secondary structures [37, 38]. Based on in silico structure prediction, this is true for *HAX1* and presumably facilitates its specific regulatory activities. Moreover, the transcript level of *hax1* was very low, suggesting that this transcript is not translated into a protein.

Another interesting point that turned out during studies on *HAX1* is its exclusive presence in *T. reesei*. This is in contrast to the main regulatory proteins of the *T. reesei* PBDE expression, which are well conserved in filamentous fungi. For example homologues of the transactivator Xyr1 have been studied and described in several other fungi such as *Neurospora crassa, Aspergillus* spp., *Fusarium* spp., or *Magnaporthe oryzae* [57, 58], and proteins resembling Cre1 in its function and sequence are even found in yeast [59]. Yet, other than for transcription factors, poor conservation is common for lncRNAs [32, 36, 60, 61]. Thus, this finding further supports the conclusion that the factor encoded by *hax1* indeed is a regulatory RNA rather than a protein.

Considering the fact that *HAX1* has an impact on PBDE expression, one outcome concerning the mechanism of *HAX1* is that it acts on distal targets rather than simply interfering with the transcriptional machinery in *cis*. Given that no targets proximal to the *hax1* locus are known to be regulated by *HAX1* strengthens this assumption. Moreover, a *trans* mode of action is supported, because ectopic overexpression of *HAX1* results in a distinct phenotype (i.e., increased cellulase activity). Both *trans* and *cis* operation modes are common for lncRNAs [32, 62]. Yet, the possibility to act on distal targets strongly extends the potential sphere of influence.

Maybe, the most remarkable finding about *HAX1* and its mechanistic strategy is that obviously different dominant versions of it are present in moderate cellulase producing and overproducing *T. reesei* strains. The *HAX1* versions differ in length, resulting from variation of the transcriptional start site. The overall longest versions of *HAX1* have been identified in the industrial progenitor strain Rut-C30. Comparing QM9414 and QM6a, again, the longer version is present in the hyper-producing strain QM9414. This fits to the different effects observed for disruption of *hax1* in QM9414 and in QM6a. In QM9414 *hax1* disrupted strains, cellulase activity was lower compared to the reference strain, whereas in QM6a, disruption of *hax1* had no phenotypic effects. Since *HAX1* versions identified in Rut-C30 are even longer, an additional enhancing effect on cellulase activity could be supposed. This assumption was supported by the overexpression of the three major *HAX1* versions (*hax1*_QM6a_, *hax1*_QM9414,_ and *hax1*_Rut-C30_) in QM6a, which led to an enhancing effect on cellulase activity with increasing *HAX1* length. Anyhow, it has to be considered that differences in RNA length impact RNA folding of *HAX1*.

A slightly similar example for production of different versions of a nc transcript is the vertebrate lncRNA *Evf*-*2*, which is generated from *Evf*-*1* by alternative splicing. For both ncRNAs, the expression is developmentally regulated; however, only *Evf*-*2* acts on neuronal differentiation in a regulatory manner by cooperating with Dlx-2 [63, 64]. In contrast to this, all three versions of *HAX1* have a functional role. The mechanism of lncRNA length variation in different strains as described herein for *T. reesei* is also remarkable on an evolutionary point of view, regarding the genesis of cellulase overproducing *T. reesei* strains like QM9414 and Rut-C30. Current data are also raising speculations about the nature of *HAX1* in industrial strains. In this regard, *HAX1* versions with greater impact on PBDE expression might be discovered. Consequently, this brings outstanding potential for utilization of *HAX1* as a tool for targeted strain improvement.

Anyway, more work has to be done to shed light on the complex strategy of alternated lncRNA production, the detailed mode of action of *HAX1* and a possible interplay with other factors involved in regulation of PBDE expression in *T. reesei*.

## Conclusion

During this study, *HAX1* was discovered and characterized as the first lncRNA in filamentous fungi. As such, *HAX1* is an outstanding example in the literature and of particular importance for basic research. Moreover, it is interesting from an evolutionary point of view as it is only present in *T. reesei*, but not in other, even closely related microorganisms. This makes *HAX1* a protruding regulatory element beside the well-conserved central transcription factors Xyr1 and Cre1. The mechanism described for *HAX1* is unique as it can result in different regulatory outputs. Based on the results presented in this study, *HAX1* can be considered as a potential target for directed engineering to promote productivity of industrially applied *T. reesei* strains.

## Methods

### Fungal strains and growth conditions

Throughout this study, the *T. reesei* strains listed in Table 1 were used. All strains were maintained on malt extract (MEX) agar at 30 °C. If applicable, 5-FOA or uridine was added to final concentrations of 1.5 mg/ml or 5 mM, respectively.

**Table 1:** Fungal strains used in this study

Name	Abbreviation	Employment	Phenotype^a^	Source
QM6a		RACE		ATCC 13631
QM6a_∆*tmus53*		Recipient strain, RACE		[70]
QM6a_∆*tmus53*_∆*pyr4*		Reference strain (∆*pyr4* background)	R/5-FOA A/uridine	[66]
QM6a_∆*tmus53*_D*hax1*_1	QM6a_D*hax1*_1	*hax1* disruption	R/amdS	This study
QM6a_∆*tmus53*_D*hax1*_2	QM6a_D*hax1*_2	*hax1* disruption	R/amdS	This study
QM6a_∆*tmus53*_D*hax1*_3	QM6a_D*hax1*_3	*hax1* disruption	R/amdS	This study
QM6a_∆*tmus53*_D*hax1*_8	QM6a_D*hax1*_8	*hax1* disruption	R/amdS	This study
QM6a_∆*tmus53*_∆*pyr4*_P*bgl1*::*hax1*_QM6a_ (deletion of *pyr4* gene)	QM6a_OE*hax1*_QM6a_	*hax1* overexpression	R/5-FOA A/uridine	This study
QM6a_∆*tmus53*_∆*pyr4*_P*bgl1*::*hax1*_QM9414_ (deletion of *pyr4* gene)	QM6a_OE*hax1*_QM9414_	*hax1* overexpression	R/5-FOA A/uridine	This study
QM6a_∆*tmus53*_∆*pyr4*_P*bgl1*::*hax1*_Rut-C30_ (deletion of *pyr4* gene)	QM6a_OE*hax1*_Rut-C30_	*hax1* overexpression	R/5-FOA A/uridine	This study
QM9414		recipient strain		ATCC 26921
QM9414_D*hax1*_8		*hax1* disruption	R/amdS	This study
QM9414_D*hax1*_10		*hax1* disruption	R/amdS	This study
QM9414_D*hax1*_14		*hax1* disruption	R/amdS	This study
QM9414_D*hax1*_8_∆*pyr4*::P*bgl1*-*hax1*_QM9414_ (deletion of *pyr4* gene)	QM9414_D*hax1*_14 _OE*hax1*_QM9414_	*hax1* overexpression	R/amdS R/5-FOA A/uridine	This study
QM9414_D*hax1*_8 _∆*pyr4*::P*bgl1*-*hax1*_Rut-C30_ (deletion of *pyr4* gene)	QM9414_D*hax1*_14 _OE*hax1*_Rut-C30_	*hax1* overexpression	R/amdS R/5-FOA A/uridine	This study
Rut-C30		RACE		ATCC 56765

^a^R/, resistance; A/, auxotrophy

For carbon source replacement experiments, mycelia were pre-cultured in 250 ml of Mandels–Andreotti (MA) medium [65] supplemented with 1% (w/v) glycerol as the sole carbon source on a rotary shaker (180 rpm) at 30 °C for 24 h. A total of 10^9^ conidia per litre (final concentration) were used as the inoculum. Pre-grown mycelia were washed, and equal amounts were resuspended in 20 ml MA medium without any carbon source (reference condition) or MA medium containing 1% (w/v) d-glucose or 1.5 mM sophorose. Samples were taken after 3 h of incubation from three biological replicates.

For direct cultivations for the β-glucosidase assays, 30 ml MA medium containing 1% (w/v) oat spelt xylan (Sigma-Aldrich, St. Louis, MO, USA) were inoculated with 10^8^ conidia per litre (final concentration) and incubated at 30 °C and 250 rpm for 66 h. For direct cultivations for cellulase assays, the D*hax1* strains were incubated for 48 h and 70 h at 30 °C and 180 rpm in 100 ml MA medium containing 1% (w/v) α-d-lactose inoculated with 10^9^ conidia per litre (final concentration). For time course experiments, the OE*hax1* strains were incubated for 72 h and 10 ml samples were taken after 37, 48, and 61 h.

### Plasmid construction

Construction of p5-A-3 used for disruption of the *hax1* locus in the *T. reesei* wild-type strain QM6a was done as follows. The 452 bp 5′- and the 1066 bp 3′-flanks of the targeted site for insertion of the *amdS* marker gene were amplified using the primers locus 5′for SalI/locus 5′rev HindIII and locus 3′for *Acc*65I/locus 3′rev *Xba*I, respectively, and cloned into pGEM-T (Promega, Madison, WI, USA). Subsequently, the 5′-fragment was released with *Hin*dIII and *Sal*I and ligated into the vector pAMDS (a derivate of pUC19 containing the 3864 bp *amdS* gene [23]) digested with the same enzymes. Finally, the 3′-fragment released with *Acc*65I and *Xba*I was introduced into the plasmid p5-A resulting from the first cloning step, downstream of the *amdS* marker gene. The final construct p5-A-3 contains the *hax1* 5′- and 3′-flanks interrupted by the *amdS* marker gene in forward orientation.

For generation of the *hax1* overexpression constructs pCD-∆pyr4-Pbgl1-hax1_QM6a_, pCD-∆pyr4-Pbgl1-hax1_QM9414_ and pCD-∆pyr4-Pbgl1-hax1_Rut-C30_, the respective *hax1* versions were amplified using chromosomal DNA of *T. reesei* QM6a as template. The following primers were used: hax1 for_QM6a_BcuI and hax1 rev_3′QM6a for the 262 bp *hax1*_QM6a_; hax1 for_QM9414_BcuI and hax1 rev_3′QM6a for the 299 bp *hax1*_QM9414_ and hax1 for_Rut-C30_BcuI and hax1 rev_3′QM6a for the 428 bp *hax1*_Rut-C30_. The purified PCR products were blunt-end ligated into pJET1.2 (Thermo Scientific, Waltham, MA, USA) and the appropriate orientation was verified by digest with BcuI and XbaI. In a next step, a 997 bp fragment of the *bgl1* promoter was PCR-amplified using the primers Pbgl1 for_Kpn2I and Pbgl1 rev-NheI, then digested with Kpn2I and NheI, and subsequently cloned into the respective pJET-hax1 vectors digested with Kpn2I and BcuI. To prevent positioning of the *hax1* gene and the foreign 5′-untranslated region of *bgl1* next to each other, the terminal point of the promoter fragment was chosen to be equal to the transcriptional start point previously defined for this gene [56]. For construction of the final *hax1* overexpression cassettes, the P*bgl1*-*hax1* fusion products were isolated from the plasmids by digestion with Kpn2I and XbaI, extracted from a gel, and introduced into BcuI/Kpn2I-digested pCD-∆pyr4 (carrying the *cbh2* terminator [66]) in forward orientation.

For cloning of the constructs, *Escherichia coli* strain Top10 (Invitrogen, Life Technologies, Paisley, UK) was used. It was maintained on LB supplemented with 100 µg/ml ampicillin or spectinomycin and grown at 37 °C. All PCRs were performed applying peqGOLD Pwo DNA polymerase (PEQLAB, Biotechnologie, Erlangen, Germany) according to the manufacturer’s instructions. The primers used are listed in Table 2. Final constructs were verified by sequencing (Microsynth, Balgach, Switzerland).

**Table 2:** Primers and probes used in this study

Name	Sequence (5′–3′)	Employment(s)
act1f	TGAGAGCGGTGGTATCCACG	qPCR
act1r	GGTACCACCAGACATGACAATGTTG	qPCR
amdS inv for	CAAAGGAAGAATCCCTTCAGGGTTGCGTTTCCAG	Inverse PCR
amdS inv rev	CGCAGTTGCGTGGGATGACATTCATACTCAAGAC	Inverse PCR
hax1 for_down-Intron	CCAGCTCCAACAGAACCAGG	Sequencing
hax for_inter-Intron	GCAATGTCGCCAGGCACCG	qPCR
hax1 for_qPCR_QM9414	GTTCAAGCCCGTTCAAGCCC	qPCR
hax1 for_QM6a_BcuI	CAGCAGTACTAGTCCCACCGGCAGGTGGCTAAACGG	Qualitative PCR, cloning OE*hax1*
hax1 for_QM9414_BcuI	CAGCAGACTAGTGGTCAGGCCCGTTCAAGCCCGTTC	Qualitative PCR, cloning OE*hax1*
hax1 for_Rut-C30_BcuI	CAAGACTAGTGAAGTTCCACACGGATACAGAGACACAACATG	Qualitative PCR, cloning OE*hax1*
hax1 for_3	TGCCTTGCATCGTACCGCTG	qPCR
hax1 for_3′RACE_3	AGGTGCTAAAACTGAATGGATGG	RACE
hax1 rev	TCCGCAAAACGAACCGAACC	qPCR
hax1 rev_down-Intron	GAGCGGCAGGAGGATGGATTC	qPCR
hax1 rev_inter-Intron	GAGGTGCGCCGCGAATCTG	Sequencing
hax1 rev kurz	TGAGTCGAGGGGCTACTGCAAGTAC	qPCR
hax1 rev_up-Intron	GCCATCCATTCAGTTTTAGCACC	qPCR, RACE
hax1 rev_1.Intron	CACGCATTTCATCTGGCCATTG	qPCR
hax1 rev_3′QM6a	CACGCATTTCATCTGGCCATTGAGTATCTACG	Qualitative PCR, cloning OE*hax1*
hax1 rev_3-39_XbaI	CAGCAGTCTAGAGACTTCTTGGTATTGTCGTCACCTTATG	Sequencing
hax1 rev_5′RACE_2	CTACGATACACCGACTTCTTGG	RACE
hax1 rev_ 5′RACE_4	CGTCCCCACAATAGCCATGAGCCG	RACE
Isopropylmalate synthase forward	TCCCGAATGCTTCTCCGACA	qPCR
Isopropylmalatesynthase_rev	GCTCTCTCTGTCTCCGATGTTGG	qPCR
Locus for	CGCGCTCTCTTTTCCTCCTT	D*hax1* candidate screening
Locus rev	GCAGAACCCAGGACACAAAGAGC	D*hax1* candidate screening
Locus 3′for *Acc*65I	GGTACCTGAGGTGTATGATTCCGTGATG	Cloning D*hax1*
Locus 3′rev XbaI	TCTAGATCCTGTTCGGCCCAGCCC	Cloning D*hax1*
Locus 5′for SalI	GGGAATAACCAATGCCTCTGAAGCTT	Cloning D*hax1*
Locus 5′rev *Hin*dIII	GGGAATAACCAATGCCTCTGAAGCTT	Cloning D*hax1*
Pbgl1 for_Kpn2I	CAAGTCCGGAGCAAGCGATAACCATAGGTA	Cloning OE*hax1*
Pbgl1 rev-NheI	CAACAAGCTAGCCTCAACAAAGCAGAGTCTTG	Cloning OE*hax1*
5pyr4_fwd(BglII)	GCGGAAGATCTCGAGATAGTATCTC	OE*hax1* candidate screening
sar1fw	TGGATCGTCAACTGGTTCTACGA	qPCR
sar1rev	GCATGTGTAGCAACGTGGTCTTT	qPCR
sonde hax_5-biotin	[biotin]CGTCAGCCACCAGCCACCGTTTAGCCACCT	*HAX1* enrichment
Unknown protein forward	CGCCGTATTGGGGTTCATTG	qPCR
Unknown protein reverse	GGCTAAACGTTGTCTCGGAG	qPCR
up-hax1 for_1	ACATGCAGGAGATTGGGCGTC	qPCR
up-hax1 for_2	CGCGGCTTAATCAGAGGTGGG	qPCR

### Fungal transformation

Accidental disruption of *hax1* (D*hax1*) in QM9414 occurred by usage of 9 µg of the vector pAMDS (a derivate of pUC19 containing the 3864 bp *amdS* gene) in an optimized protocol for particle bombardment [67]. Selection for positive transformants carrying the *amdS* gene, was performed on minimal medium (72.9 mM KH_2_PO_4_, 10.2 mM sodium citrate, 1% (w/v) d-glucose, 20 ml/L trace element solution (see MA medium [65]), and 1.5% agar noble dissolved in ultrapure H_2_O) containing acetamide. After autoclaving, acetamide, CsCl and MgSO_4_ were added from sterile-filtered stock solutions to final concentrations of 10, 10, and 24.3 mM, respectively.

Protoplast transformation of *T. reesei* was performed as described previously [68]. QM6a_D*hax1* strains resulted from transformation of 13 µg *hax1* 5′-*amdS*-3′ DNA derived from a *Hin*dIII/*Acc*65I-digest of p5-A-3 and extraction from a gel. The template DNA was resuspended in 15 µl sterile dH_2_O and used for transformation of 10^7^ protoplasts (in 150 μl) of QM6a_∆*tmus53*. 100 μl–2 ml of the transformation reaction were added to 20 ml melted, 50 °C warm *amdS* selection medium supplemented with 1.2 M sorbitol. This mixture was poured into sterile petri dishes.

For overexpression of *hax1* (OE*hax1*), 200 µg of the *Not*I-digested construct pCD-∆pyr4-Pbgl1-hax1_QM6a_, pCD-∆pyr4-Pbgl1-hax1_QM9414,_ or pCD-∆pyr4-Pbgl1-hax1_Rut-C30_ were used for transformation of 10^7^ protoplasts (in 150 μl) of QM9414_D*hax1* or QM6a_∆*tmus53*. Selection for *pyr4* deleted transformants was performed on MEX agar containing 1.2 M sorbitol, 1.5 mg/ml 5-FOA, and 5 mM uridine as described by Derntl and co-workers [66].

Plates were incubated at 30 °C for 3–7 days until colonies were visible.

### Genotypic characterization

For an initial identification of uridine auxotroph OE*hax1* strains, candidates were grown on MA medium containing 1% glycerol as a carbon source without peptone or uridine. The OE*hax1* candidates that were unable to grow under these conditions were tested by PCR using the primers 5pyr4_fwd(BglII) and hax1 rev_3′QM6a.

For D*hax1* strains, homokaryotic strains were generated by three rounds of vegetative spore propagation on selection medium. In case of QM9414_D*hax1* strains, the locus of integration was identified via inverse PCR and verified by Southern blot analysis. In case of QM6a_D*hax1* strains correct integration of the construct was tested by PCR and by sequencing.

Extraction of chromosomal DNA for candidate screening and Southern blot analysis was performed as described previously [65, 66]. For Southern blot analysis, the chromosomal DNA of QM9414_D*hax1* strains was digested with SacII, resulting in a 2960 bp fragment specific for the wild type and a 5178 bp fragment specific for disruption of *hax1*. The locus-specific biotinylated probe applied for hybridization was derived from a PCR using the primer pair locus for and locus rev and the Long-PCR Enzyme Mix (Thermo Scientific) for amplification.

PCR analysis for screening for D*hax1* candidates was performed using the same polymerase and primers. For verification of OE*hax1* transformants via PCR, GoTaq G2 polymerase (Promega) was applied. DNA sequencing was performed at Microsynth. All primers used for candidate screening are listed in Table 2.

### Inverse PCR

For the identification of the locus that was targeted by integration of the *amdS* marker in QM9414_D*hax1* strains, an inverse PCR was performed as follows. 20 µg of chromosomal DNA were digested with either *Acc*65I or *Not*I (Thermo Scientific) at a final concentration of 1 U/µl in a total reaction volume of 20 µl according to the manufacturer’s instructions. After heat inactivation, 2 µl of T4 Ligase (Promega, 1–3 U/µl) and the corresponding buffer were added and ligation was performed at 18 °C for 90 min. Subsequently, the ligation was stopped by heat inactivation and 1 µl was applied as template in a 25 µl inverse PCR reaction, initially using the primers amdS inv for and amdS inv rev. For further approaches, the primers locus for and locus rev annealing to the identified regions were applied. All amplifications were performed in an iCycler (Bio-Rad, Hercules, CA, USA) using the Long-PCR Enzyme Mix (Thermo Scientific) and the following program: initial denaturation at 94 °C for 3 min, followed by 30 cycles of 30 s at 94 °C, 30 s at 59 °C and 3 min at 72 °C, and final elongation at 72 °C for 5 min. The DNA fragments were sequenced by MWG Biotech (Ebersberg, Germany).

### RNA stability assay

The strains QM6a, QM9414, and Rut-C30 were cultivated in 25 ml MA medium containing 1% (w/v) α-d-lactose and 10^9^ conidia per litre (final concentration) at 30 °C and 180 rpm for 24 h. The cultivation was carried out in biological triplicates. Then, the transcription was stopped by addition of 16 µg/ml (final concentration) of the inhibitor DRB. The cultures were further incubated and after 30, 60, 90, and 120 min, 500 µl samples were taken. A reference sample was taken from each culture before addition of DRB. The three biological replicates of each strain were pooled to one 1.5 ml sample. Mycelia were harvested by centrifugation and frozen in liquid nitrogen. Finally, the RNA was extracted, cDNA synthesis was performed and the samples were analysed via quantitative PCR as described in “Transcript analysis” section.

### Transcript analysis

Extraction of RNA from fungal mycelia, cDNA synthesis, and quantitative PCRs were performed as described previously [69]. Template cDNAs were diluted 1:100 (QM9414) or 1:20 (QM6a). Analysis was carried out in triplicates. The following PCR protocols were run: 3 min initial denaturation at 95 °C, followed by 50 cycles of 15 s at 95 °C, 15 s at 60 °C and 20 s at 72 °C (for *hax1* and *act*) or 3 min initial denaturation at 95 °C, followed by 40 cycles of 15 s at 95 °C and 120 s at 64 °C (for *sar1*). Control reactions, data normalization using *sar1* and *act* as reference genes and calculations were performed as published previously [70].

Qualitative PCRs for estimating the abundance of *hax1*_QM6a_, *hax1*_QM9414,_ and *hax1*_Rut-C30_ were performed using the Q5 High-Fidelity DNA Polymerase (New England Biolabs, Ipswich, MA, USA) according to the manufacturer’s instructions. The template cDNAs were diluted 1:20. Amplification was performed running the following program: initial denaturation at 98 °C for 30 s, followed by 35 cycles of 10 s at 98 °C, 10 s at 70 °C and 20 s at 72 °C, and final elongation at 72 °C for 2 min. The resulting fragments (262, 299, and 428 bp) were analysed on a 1.5% agarose gel applying a GeneRuler 50 bp DNA Ladder (Thermo Scientific) for size estimation. Primer sequences for all transcript analyses are provided in Table 2.

### RACE and enrichment of *HAX1*

5′ and 3′ RACE was performed using the 5′/3′ RACE Kit, 2nd generation (Roche, Basel, Switzerland). For PCRs, the GoTaq G2 polymerase (Promega) was applied. Primer sequences for RACE are provided in Table 2.

5′ RACE was carried out according to manufacturer’s instructions, applying 0.9–1 µg of DNase I digested RNA extracts for cDNA synthesis in a total reaction volume of 20 µl. For this initial reverse transcription step, the gene specific primer hax1 rev_1.Intron was used. After RNase A digestion and purification with the QIAquick PCR Purification Kit (Qiagen, Hilden, Germany) based on the modified protocol for RACE applications recommended by Roche, a poly(A)-tailing was performed. Subsequently, the *hax1* fragments were specifically amplified from the total pool with the Oligo dT-Anchor Primer (included in the kit) and hax1 rev_5′RACE_2 in a first PCR. The resulting product was diluted 1:50 and used as a template for a nested PCR with either hax1 rev_up-Intron or hax1 rev_ 5′RACE_4 and the PCR Anchor primer included in the kit.

For 3′ RACE, classical analysis was performed according to manufacturer’s instructions, applying 0.45 µg of DNase I digested RNA from *T. reesei* QM9414 in a total reaction volume of 20 µl. After reverse transcription with the Oligo dT-Anchor Primer, cDNAs were RNase A digested and used for amplification of *hax1* applying the gene specific primers up hax1 for_2 and hax1 for_3′RACE_3 for the initial and nested PCRs, respectively. Further 3′ RACE approaches based on prior enrichment of *HAX1* were performed. For this purpose, *HAX1* was enriched from 1.25 to 2.1 mg total RNA extract using a biotinylated and HPLC-purified *hax1* specific DNA probe (sonde hax_5-Biotin) as well as streptavidin-linked magnetic beads included in the µMACS™ Streptavidin Kit and the corresponding µMACS separator (Miltenyi Biotec, Bergisch Gladbach, Germany) based on manufacturer’s instructions. According to the unusual high calculated melting temperature of the biotinylated probe, initial denaturation was performed at 85 °C for 5 min and annealing to appropriate amounts of streptavidin-linked magnetic beads was done at 70 °C for 15 min. RNase-free TEN buffer (10 mM Tris/HCl, pH 8.0; 1 mM EDTA; 100 mM NaCl_2_) and TE buffer (10 mM Tris/HCl, pH 8.0; 1 mM EDTA) were used for binding and washing, respectively. Enriched RNA was eluted with 150 µl RNase-free dH_2_O and digested with DNase I (Thermo Scientific). For poly(A)-tailing followed by RACE analysis, the enriched RNA was precipitated with isopropanol, washed with 70% (w/v) ethanol and dissolved in 20 µl RNase-free dH_2_O. Poly(A)-tailing was performed using 19 µl of the resulting RNA in a total reaction volume of 25 µl. For proceeding to cDNA synthesis without prior poly(A)-tailing, the RNA was purified using the GeneJET RNA Cleanup and Concentration Micro Kit (Thermo Scientific) as instructed in the protocol for purification of DNase I digested samples. In this case, the RNA was eluted from four columns with 10 µl each and pooled for cDNA synthesis. Reverse transcription and further 3′ RACE analysis steps were performed as described before using the RACE Kit and applying the gene specific primers up hax1 for_2 and hax1 for_3′RACE_3 for the initial and nested PCRs, respectively.

For both, 3′ and 5′ RACE, the final PCR products were extracted from a gel, blunt-end ligated into pJET1.2 (Thermo Scientific) and analysed by sequencing (Microsynth).

### Enzyme assays

The β-glucosidase activity was assayed in 50 mM sodium citrate buffer at pH 5.0 using *p*-nitrophenyl-β-d-glucopyranoside as a substrate. The enzyme assay was performed at 50 °C for 30 min as previously described [71] and the activity was calculated from the absorbance at 405 nm based on the Lambert–Beer law.

The cellulase activity was assayed in 25 mM sodium acetate buffer pH 4.5 using Azo-Cellazyme C tablets (Megazyme, Wicklow, Ireland) as substrate, essentially according to the manufacturer’s instructions. The reaction time was increased to 30 min–5 h until detectable values were obtained. The same reaction times were used for all strains cultivated for the same time period within one experiment, and samples with higher cellulase activity were adjusted by dilution to enable comparison. Cellulase activities given in μ were calculated from the absorbance at 590 nm for 10 min reaction time based on the equation μ = 232.6 * Abs + 5 [72].

One unit is defined as the amount of enzyme required to release 1 µmol of d-glucose reducing-sugar-equivalents per minute under the respective assay conditions. For the final timepoints of cultivation, the enzymatic activities were referred to the biomass dry weight derived from incubation of the harvested mycelia at 80 °C for 24 h.

### Structure and sequence analysis tools

Genome analysis and BLAST were performed using the NCBI database [54] and the sequence of *Trichoderma reesei* QM6a v2.0 accessible in the JGI database [51]. For structural gene prediction, the web server AUGUSTUS described by Stank and Morgenstern was used [52]. Codon usage was calculated applying the online Codon Usage Calculator of Biologics Corp [53]. In silico prediction of RNA secondary structures of minimized free energy was performed using the RNAfold Web server [55] included in the ViennaRNA Package 2.0 [73], provided by the University of Vienna. Displayed structures are based on *HAX1* sequences without polyA-tail; however, the addition of a random polyA-tail did not change structures.

### Statistical test

For statistical analyses of the data, the program GraphPad Prism 5.00.288 was used to perform ANOVA (*P* < 0.05) tests and Tukey’s multiple comparison test as posttest.

## Additional files

**Additional file 1.** Transcript levels of the neighbouring genes of the targeted site for *amdS* integration. RT-qPCR results of an undescribed protein referred to as hypothetical protein (Protein ID 108999) (**A**) and the 2-isopropylmalate synthase (Protein ID 79495) (**B**) in *T. reesei* strains QM9414 (PS; black bars), QM9414_D*hax1*_8 (D8; orange bars) and QM9414_D*hax1*_10 (D10; red bars). cDNAs used as templates were derived from cultures that were pre-grown and transferred to medium without carbon source, 1% (w/v) d-glucose (G) or 1.5 mM sophorose (S) for 3 h. Transcript levels were normalized to *act* and *sar1,* refer to QM9414 (no carbon source) and are given in logarithmic scale (lg). Analysis was performed in technical triplicates. The error bars depict the standard deviation and different letters denote statistical difference among compared data employing ANOVA (*P* < 0.05).

**Additional file 2.** Codon usage of the predicted *hax1* gene. For each of the 64 codons potentially making up a protein the following information is listed: base triplet; the encoded amino acid (given as one-letter code); fraction out of the 313 codons constituting *hax1*; frequency per thousand; absolute number of occurrence (from a total of 313 codons). Asterisks indicate stop codons.

**Additional file 3.** Curves of RT-qPCR for the detection of the *hax1* transcript in *T. reesei* QM6a. *T. reesei* QM6a was cultivated on 1% d-glucose (brown) or without carbon source (blue). The qPCR was performed with primer pair up-hax1 for_2 and hax1 rev 1.Intron (compare Fig. 2c, PCR 2). Analysis was performed in technical triplicates. A no template control (red) and a negative control (yellow) were included in the run. Measured fluorescence is plotted against the number PCR cycles.

**Additional file 4.** Alignment of the *hax1* locus of the investigated *T. reesei* strains. The *hax1* locus (letters without background) and the adjacent regions encoding the neighbouring genes (letters on grey background) of different *T. reesei* strains were PCR-amplified from chromosomal DNA using the primers locus for and locus rev and sequenced using the primers hax1 rev_inter-Intron, hax1 rev_3-39_XbaI, up-hax1 for_2 and hax1 for_down-Intron. Assembled sequences determined for Rut-C30 (purple letters) and QM9414 (green letters) were aligned to the genome of *T. reesei* QM6a (black, bold, underlined letters) accessible in the Joint Genome Institute database. The whole investigated sequence revealed 100% identity.

**Additional file 5.** RNA stability of the three *HAX1 versions*. The *T. reesei* strains QM6a (blue), QM9414 (yellow) and Rut-C30 (purple) were grown in MA medium containing 1% (w/v) α-d-lactose for 24 h and transcription was inhibited by the addition of DRB. Samples were taken before the addition of DRB (reference sample) and 30 min, 60 min, 90 min and 120 min after the addition of DRB. RT-qPCR was performed using cDNA from QM6a for the amplification of *hax1*_QM6a_ (primer pair up-hax1 for_2 and hax1 rev_1.Intron, blue), from QM9414 for the amplification of *hax1*_QM9414_ (primer pair hax1 for_qPCR_QM9414 and hax1 rev_up-Intron; yellow) and from Rut-C30 for the amplification of *hax1*_Rut-C30_ (primer pair up-hax1 for_1 and hax1 rev_up-Intron, purple). Transcript levels were normalized to *act* and *sar1*, refer to the sample taken before the addition of DRB (time point 0 min) and are given in logarithmic scale (lg). All standard deviations of technical triplicates are < 0.06.

**Additional file 6.** RT-qPCR of the three *hax1* versions in QM9414 disruption strains. Analysis of the formation of *hax1* transcripts, i.e., *hax1*_QM6a_ (graph on top), *hax1*_QM9414_ (graph in the middle) and *hax1*_Rut-C30_ (graph at the bottom), in QM9414_D*hax1*_8 (green) and QM9414_D*hax1*_14 (blue) and their parent strain QM9414 as a positive control (grey). The cDNAs used as templates were derived from cultivation on lactose for 48 h (QM9414_D*hax1* strains) or replacement to medium without carbon source (positive control). For amplification the primer pairs up-hax1 for_2 and hax1 rev_1.Intron (*hax1*_QM6a_), hax1 for_qPCR_QM9414 and hax1 rev_up-Intron (*hax1*_QM9414_), or up-hax1 for_1 and hax1 rev_up-Intron (*hax1*_Rut-C30_) were used. Analysis was performed in technical triplicates. A no template control (red) and a negative control (yellow) were included in each run. Measured fluorescence is plotted against the number PCR cycles.
