# Supplementary figures and images for: A long noncoding RNA promotes cellulase expression in Trichoderma reesei
Source: Biotechnol Biofuels. 2018 Mar 23;11:78. doi: 10.1186/s13068-018-1081-4 (PMC5865335; doi:10.1186/s13068-018-1081-4)

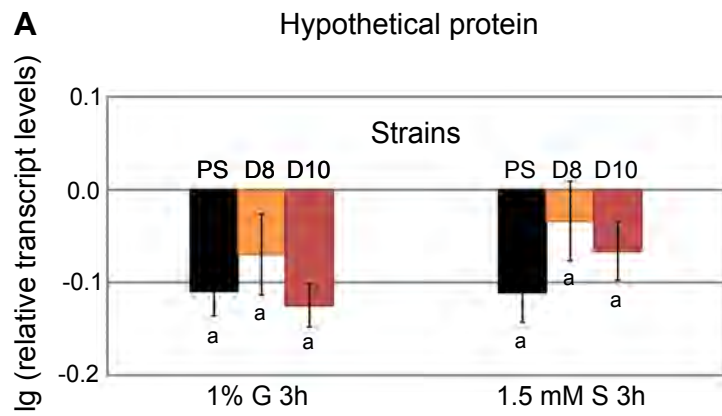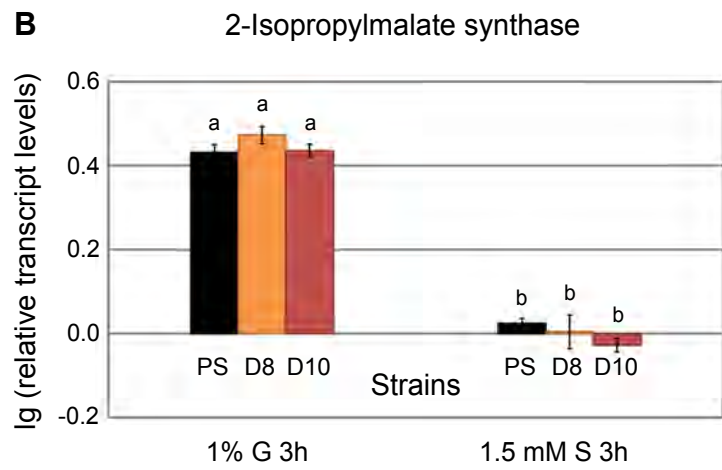

Supplement: Supplementary file 1 — Additional file 1. Transcript levels of the neighbouring genes of the targeted site for amdS integration. RT-qPCR results of an undescribed protein referred to as hypothetical protein (Protein ID 108999) (A) and the 2-isopropylmalate synthase (Protein ID 79495) (B) in T. reesei strains QM9414 (PS; black bars), QM9414_Dhax1_8 (D8; orange bars) and QM9414_Dhax1_10 (D10; red bars). cDNAs used as templates were derived from cultures that were pre-grown and transferred to medium without carbon source, 1% (w/v) d-glucose (G) or 1.5 mM sophorose (S) for 3 h. Transcript levels were normalized to act and sar1, refer to QM9414 (no carbon source) and are given in logarithmic scale (lg). Analysis was performed in technical triplicates. The error bars depict the standard deviation and different letters denote statistical difference among compared data employing ANOVA (P < 0.05). [file 13068_2018_1081_MOESM1_ESM.pdf]

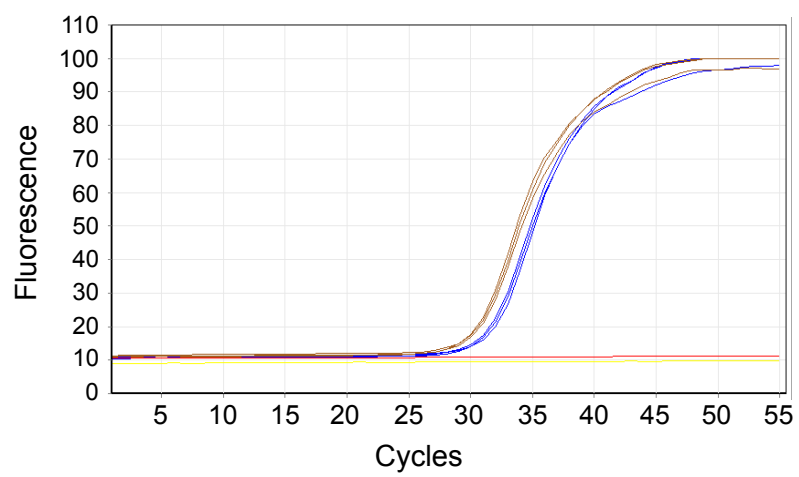

Supplement: Supplementary file 3 — Additional file 3. Curves of RT-qPCR for the detection of the hax1 transcript in T. reesei QM6a. T. reesei QM6a was cultivated on 1% d-glucose (brown) or without carbon source (blue). The qPCR was performed with primer pair up-hax1 for_2 and hax1 rev 1.Intron (compare Fig. 2c, PCR 2). Analysis was performed in technical triplicates. A no template control (red) and a negative control (yellow) were included in the run. Measured fluorescence is plotted against the number PCR cycles. [file 13068_2018_1081_MOESM3_ESM.pdf]

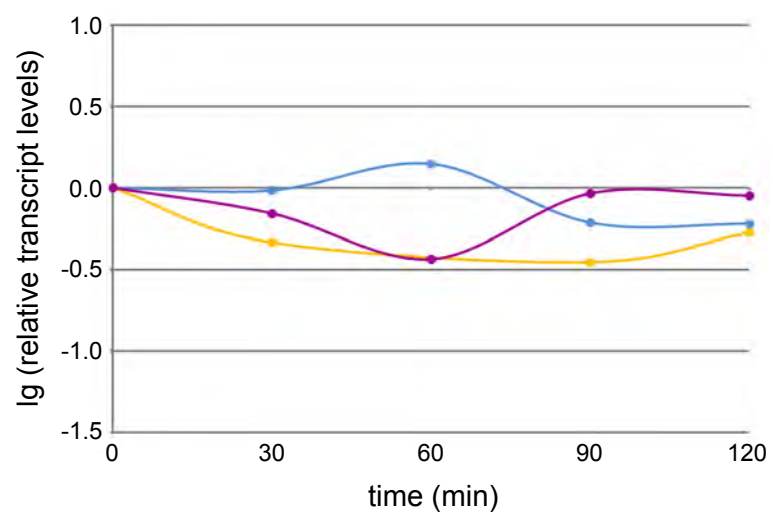

Supplement: Supplementary file 5 — Additional file 5. RNA stability of the three HAX1 versions. The T. reesei strains QM6a (blue), QM9414 (yellow) and Rut-C30 (purple) were grown in MA medium containing 1% (w/v) α-d-lactose for 24 h and transcription was inhibited by the addition of DRB. Samples were taken before the addition of DRB (reference sample) and 30 min, 60 min, 90 min and 120 min after the addition of DRB. RT-qPCR was performed using cDNA from QM6a for the amplification of hax1QM6a (primer pair up-hax1 for_2 and hax1 rev_1.Intron, blue), from QM9414 for the amplification of hax1QM9414 (primer pair hax1 for_qPCR_QM9414 and hax1 rev_up-Intron; yellow) and from Rut-C30 for the amplification of hax1Rut-C30 (primer pair up-hax1 for_1 and hax1 rev_up-Intron, purple). Transcript levels were normalized to act and sar1, refer to the sample taken before the addition of DRB (time point 0 min) and are given in logarithmic scale (lg). All standard deviations of technical triplicates are < 0.06. [file 13068_2018_1081_MOESM5_ESM.pdf]

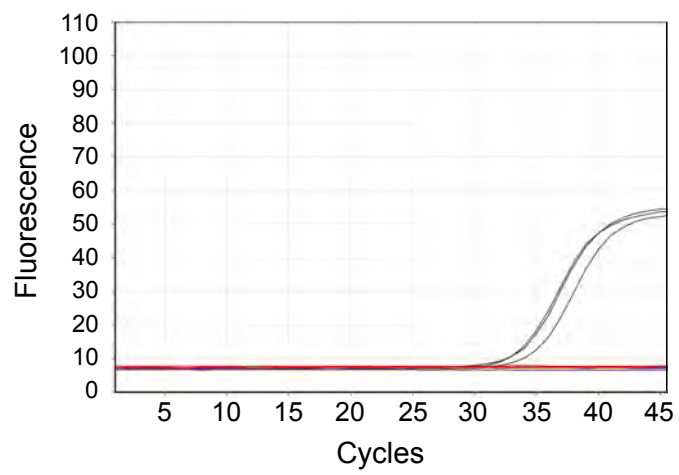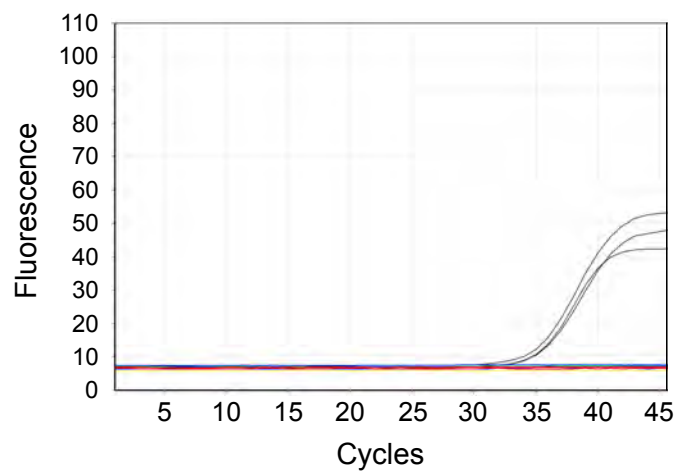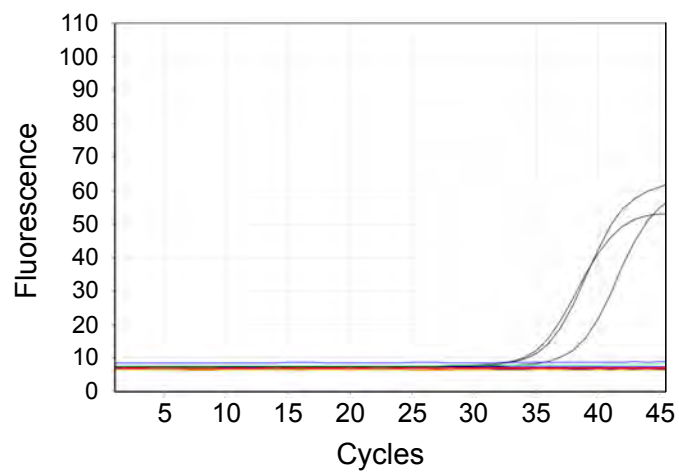

Supplement: Supplementary file 6 — Additional file 6. RT-qPCR of the three hax1 versions in QM9414 disruption strains. Analysis of the formation of hax1 transcripts, i.e., hax1QM6a (graph on top), hax1QM9414 (graph in the middle) and hax1Rut-C30 (graph at the bottom), in QM9414_Dhax1_8 (green) and QM9414_Dhax1_14 (blue) and their parent strain QM9414 as a positive control (grey). The cDNAs used as templates were derived from cultivation on lactose for 48 h (QM9414_Dhax1 strains) or replacement to medium without carbon source (positive control). For amplification the primer pairs up-hax1 for_2 and hax1 rev_1.Intron (hax1QM6a), hax1 for_qPCR_QM9414 and hax1 rev_up-Intron (hax1QM9414), or up-hax1 for_1 and hax1 rev_up-Intron (hax1Rut-C30) were used. Analysis was performed in technical triplicates. A no template control (red) and a negative control (yellow) were included in each run. Measured fluorescence is plotted against the number PCR cycles. [file 13068_2018_1081_MOESM6_ESM.pdf]
