# Supplementary material for: A long noncoding RNA promotes cellulase expression in Trichoderma reesei
Source: Biotechnol Biofuels. 2018 Mar 23;11:78. doi: 10.1186/s13068-018-1081-4 (PMC5865335; doi:10.1186/s13068-018-1081-4)
Supplement: Supplementary file 2 — Additional file 2. Codon usage of the predicted hax1 gene. For each of the 64 codons potentially making up a protein the following information is listed: base triplet; the encoded amino acid (given as one-letter code); fraction out of the 313 codons constituting hax1; frequency per thousand; absolute number of occurrence (from a total of 313 codons). Asterisks indicate stop codons. [file 13068_2018_1081_MOESM2_ESM.pdf]

Fields: [triplet] [amino acid] [frequency: per thousand] ([number])

TTT	F	0.25	4.7 (1)	TCT	S	0.00	0.0 (0)	TAT	Y	1.00	0.0 (2)	TGT	C	0.40	9.4 (2)
TTC	F	0.75	14.2 (3)	TCC	S	0.27	28.3 (6)	TAC	Y	0.00	0.0 (0)	TGC	C	0.60	14.2 (3)
TTA	L	0.00	0.0 (0)	TCA	S	0.14	14.2 (3)	TAA	*	0.00	0.0 (0)	TGA	*	1.00	4.7 (1)
TTG	L(s)	0.16	14.2 (3)	TCG	S	0.41	42.5 (9)	TAG	*	0.00	0.0 (0)	TGG	W	1.00	28.3 (6)
CTT	L	0.16	14.2 (3)	CCT	P	0.23	28.3 (6)	CAT	H	0.44	18.9 (4)	CGT	R	0.05	4.7 (1)
CTC	L	0.47	42.5 (9)	CCC	P	0.38	47.2 (10)	CAC	H	0.56	23.6 (5)	CGC	R	0.43	42.5 (9)
CTA	L	0.11	9.4 (2)	CCA	P	0.23	28.3 (6)	CAA	Q	0.38	14.2 (3)	CGA	R	0.19	18.9 (4)
CTG	L(s)	0.11	9.4 (2)	CCG	P	0.15	18.9 (4)	CAG	Q	0.62	23.6 (5)	CGG	R	0.14	14.2 (3)
ATT	I	0.20	4.7 (1)	ACT	T	0.22	9.4 (2)	AAT	N	0.33	4.7 (1)	AGT	S	0.05	4.7 (1)
ATC	I	0.80	18.9 (4)	ACC	T	0.56	23.6 (5)	AAC	N	0.67	9.4 (2)	AGC	S	0.14	14.2 (3)
ATA	I	0.00	0.0 (0)	ACA	T	0.11	4.7 (1)	AAA	K	0.60	14.2 (3)	AGA	R	0.10	9.4 (2)
ATG	M(s)	1.00	9.4 (2)	ACG	T	0.11	4.7 (1)	AAG	K	0.40	9.4 (2)	AGG	R	0.10	9.4 (2)
GTT	V	0.38	14.2 (3)	GCT	A	0.35	61.3 (13)	GAT	D	1.00	9.4 (2)	GGT	G	0.33	18.9 (4)
GTC	V	0.13	4.7 (1)	GCC	A	0.22	37.7 (8)	GAC	D	0.00	0.0 (0)	GGC	G	0.33	18.9 (4)
GTA	V	0.25	9.4 (2)	GCA	A	0.30	51.9 (11)	GAA	E	0.67	18.9 (4)	GGA	G	0.25	14.2 (3)
GTG	V	0.25	9.4 (2)	GCG	A	0.14	23.6 (5)	GAG	E	0.33	9.4 (2)	GGG	G	0.08	4.7 (1)
