# Supplementary material for: A long noncoding RNA promotes cellulase expression in Trichoderma reesei
Source: Biotechnol Biofuels. 2018 Mar 23;11:78. doi: 10.1186/s13068-018-1081-4 (PMC5865335; doi:10.1186/s13068-018-1081-4)
Supplement: Supplementary file 4 — Additional file 4. Alignment of the hax1 locus of the investigated T. reesei strains. The hax1 locus (letters without background) and the adjacent regions encoding the neighbouring genes (letters on grey background) of different T. reesei strains were PCR-amplified from chromosomal DNA using the primers locus for and locus rev and sequenced using the primers hax1 rev_inter-Intron, hax1 rev_3-39_XbaI, up-hax1 for_2 and hax1 for_down-Intron. Assembled sequences determined for Rut-C30 (purple letters) and QM9414 (green letters) were aligned to the genome of T. reesei QM6a (black, bold, underlined letters) accessible in the Joint Genome Institute database. The whole investigated sequence revealed 100% identity. [file 13068_2018_1081_MOESM4_ESM.pdf]

```
Fri Nov 17, 2017 20:05 CET
QM6a_short.ape from 1 to 2098
Alignment to
QM9414_seq.ape--  Matches:2098; Mismatches:0; Gaps:0; Unattempted:0
RutC30_seq.ape--  Matches:2098; Mismatches:0; Gaps:0; Unattempted:0

                 *         *         *         *         *         *         *         *         *         *
  1>TACAAGGAACACGCCATTGGAGAGGGTCGTGGTGTGAAGGCGGCTTCCTACTTGGAGTGCAAGCCCACGGGCAGCAAACAGACGGTCTGGGGCGTTGGCA>100
  1>TACAAGGAACACGCCATTGGAGAGGGTCGTGGTGTGAAGGCGGCTTCCTACTTGGAGTGCAAGCCCACGGGCAGCAAACAGACGGTCTGGGGCGTTGGCA>100
  1>TACAAGGAACACGCCATTGGAGAGGGTCGTGGTGTGAAGGCGGCTTCCTACTTGGAGTGCAAGCCCACGGGCAGCAAACAGACGGTCTGGGGCGTTGGCA>100

                 *         *         *         *         *         *         *         *         *         *
101>TTCACGAAGATGTGGTGTACAGCTCGCTCCTGGCCATGCTCAGTGCGGCAAGCAACGTAAGTTCTCACATGCATCGATGGTAGTAGTACCTTGTGAGTTG>200
101>TTCACGAAGATGTGGTGTACAGCTCGCTCCTGGCCATGCTCAGTGCGGCAAGCAACGTAAGTTCTCACATGCATCGATGGTAGTAGTACCTTGTGAGTTG>200
101>TTCACGAAGATGTGGTGTACAGCTCGCTCCTGGCCATGCTCAGTGCGGCAAGCAACGTAAGTTCTCACATGCATCGATGGTAGTAGTACCTTGTGAGTTG>200

                 *         *         *         *         *         *         *         *         *         *
201>ATGGCTGACGACGGTGCTTTTCGCATTTACAGTTCTTGACCAGCAGACCCACCAGCCCTCTGCTGAAGCCTGTTGCAAATCCCCCCTCACAGGAGACGCC>300
201>ATGGCTGACGACGGTGCTTTTCGCATTTACAGTTCTTGACCAGCAGACCCACCAGCCCTCTGCTGAAGCCTGTTGCAAATCCCCCCTCACAGGAGACGCC>300
201>ATGGCTGACGACGGTGCTTTTCGCATTTACAGTTCTTGACCAGCAGACCCACCAGCCCTCTGCTGAAGCCTGTTGCAAATCCCCCCTCACAGGAGACGCC>300

                 *         *         *         *         *         *         *         *         *         *
301>TAATATCGTCTCCGTTCTGGAGGAAAAGGCAAATGGTGTTTAGGGTGCTGAAACTCAAAGCAGAGGCATTGGTTATTCCCCCTTCGTATATCTAAAAGTT>400
301>TAATATCGTCTCCGTTCTGGAGGAAAAGGCAAATGGTGTTTAGGGTGCTGAAACTCAAAGCAGAGGCATTGGTTATTCCCCCTTCGTATATCTAAAAGTT>400
301>TAATATCGTCTCCGTTCTGGAGGAAAAGGCAAATGGTGTTTAGGGTGCTGAAACTCAAAGCAGAGGCATTGGTTATTCCCCCTTCGTATATCTAAAAGTT>400

                 *         *         *         *         *         *         *         *         *         *
401>CTTCGTTGCATCACCCCAAGCAGATCAACTGGCATGGGGCAGTTTTCCTTCATTTCAGAGAAGTGGACGACGAACATGACTTATCATGCGTAGCTCAGGG>500
401>CTTCGTTGCATCACCCCAAGCAGATCAACTGGCATGGGGCAGTTTTCCTTCATTTCAGAGAAGTGGACGACGAACATGACTTATCATGCGTAGCTCAGGG>500
401>CTTCGTTGCATCACCCCAAGCAGATCAACTGGCATGGGGCAGTTTTCCTTCATTTCAGAGAAGTGGACGACGAACATGACTTATCATGCGTAGCTCAGGG>500

                 *         *         *         *         *         *         *         *         *         *
501>CTGCGTACAGATAGTAAGAAGTTCCACACGGATACAGAGACACAACATGCAGGAGATTGGGCGTCATCCTTGTTCCACACGTTTTCTACCTAGGTAGCTG>600
501>CTGCGTACAGATAGTAAGAAGTTCCACACGGATACAGAGACACAACATGCAGGAGATTGGGCGTCATCCTTGTTCCACACGTTTTCTACCTAGGTAGCTG>600
501>CTGCGTACAGATAGTAAGAAGTTCCACACGGATACAGAGACACAACATGCAGGAGATTGGGCGTCATCCTTGTTCCACACGTTTTCTACCTAGGTAGCTG>600
```

```
               *         *         *         *         *         *         *         *         *         *
601>TAACAACAAGATTTACAACCAGAGCCCGAAGCTGTTCCTTGGTGATGGTCAGGCCCGTTCAAGCCCGTTCAAGCCCGTCCAAACCCACCGGCAGGTGGCT>700
601>TAACAACAAGATTTACAACCAGAGCCCGAAGCTGTTCCTTGGTGATGGTCAGGCCCGTTCAAGCCCGTTCAAGCCCGTCCAAACCCACCGGCAGGTGGCT>700
601>TAACAACAAGATTTACAACCAGAGCCCGAAGCTGTTCCTTGGTGATGGTCAGGCCCGTTCAAGCCCGTTCAAGCCCGTCCAAACCCACCGGCAGGTGGCT>700

               *         *         *         *         *         *         *         *         *         *
701>AAACGGTGGCTGGTGGCTGACGCCCGCGGCTTAATCAGAGGTGGGAGCTACTTAGCAGTCAGACAAAGCCGAGATGGCGTCGAACGGCTCATGGCTATTG>800
701>AAACGGTGGCTGGTGGCTGACGCCCGCGGCTTAATCAGAGGTGGGAGCTACTTAGCAGTCAGACAAAGCCGAGATGGCGTCGAACGGCTCATGGCTATTG>800
701>AAACGGTGGCTGGTGGCTGACGCCCGCGGCTTAATCAGAGGTGGGAGCTACTTAGCAGTCAGACAAAGCCGAGATGGCGTCGAACGGCTCATGGCTATTG>800

               *         *         *         *         *         *         *         *         *         *
801>TGGGGACGCAGCTCCTGTTCGGCCCAGCCCGCAGGTGCTAAAACTGAATGGATGGCTGGGAGAGAAGAAGTCGAGAACCATAAGGTGACGACAATACCAA>900
801>TGGGGACGCAGCTCCTGTTCGGCCCAGCCCGCAGGTGCTAAAACTGAATGGATGGCTGGGAGAGAAGAAGTCGAGAACCATAAGGTGACGACAATACCAA>900
801>TGGGGACGCAGCTCCTGTTCGGCCCAGCCCGCAGGTGCTAAAACTGAATGGATGGCTGGGAGAGAAGAAGTCGAGAACCATAAGGTGACGACAATACCAA>900

               *         *         *         *         *         *         *         *         *         *
901>GAAGTCGGTGTATCGTAGATACTCAATGGCCAGATGAAATGCGTGTAAATGATTACATACTTCCGTACCTTAGGCTGTGGTGCAATGTCGCCAGGCACCG>1000
901>GAAGTCGGTGTATCGTAGATACTCAATGGCCAGATGAAATGCGTGTAAATGATTACATACTTCCGTACCTTAGGCTGTGGTGCAATGTCGCCAGGCACCG>1000
901>GAAGTCGGTGTATCGTAGATACTCAATGGCCAGATGAAATGCGTGTAAATGATTACATACTTCCGTACCTTAGGCTGTGGTGCAATGTCGCCAGGCACCG>1000

                *         *         *         *         *         *         *         *         *         *
1001>GCGCTGGGAAGCAGATTCGCGGCGCACCTCGCACCACCACTAGGTCAACACCAGCCTCATCGAACCTGTACTCCTCCATTATTATTACTCCATACTCCAG>1100
1001>GCGCTGGGAAGCAGATTCGCGGCGCACCTCGCACCACCACTAGGTCAACACCAGCCTCATCGAACCTGTACTCCTCCATTATTATTACTCCATACTCCAG>1100
1001>GCGCTGGGAAGCAGATTCGCGGCGCACCTCGCACCACCACTAGGTCAACACCAGCCTCATCGAACCTGTACTCCTCCATTATTATTACTCCATACTCCAG>1100

                *         *         *         *         *         *         *         *         *         *
1101>CTCCAACAGAACCAGGAATCCATCCTCCTGCCGCTCTGCTCAAAGTACTTGCAGTAGCCCCTCGACTCATCCAGCACGCTCAGCTCCGCCCCGTTGCACT>1200
1101>CTCCAACAGAACCAGGAATCCATCCTCCTGCCGCTCTGCTCAAAGTACTTGCAGTAGCCCCTCGACTCATCCAGCACGCTCAGCTCCGCCCCGTTGCACT>1200
1101>CTCCAACAGAACCAGGAATCCATCCTCCTGCCGCTCTGCTCAAAGTACTTGCAGTAGCCCCTCGACTCATCCAGCACGCTCAGCTCCGCCCCGTTGCACT>1200

                *         *         *         *         *         *         *         *         *         *
1201>CCCCGGCTCCTCAGCCGCAGCACATGCGCGGCCTCTCTCGCGCGGTGCCTTGCATCGTACCGCTGCTCGAGCATCAAAACCTCCAGCCACCCAATATTAT>1300
1201>CCCCGGCTCCTCAGCCGCAGCACATGCGCGGCCTCTCTCGCGCGGTGCCTTGCATCGTACCGCTGCTCGAGCATCAAAACCTCCAGCCACCCAATATTAT>1300
1201>CCCCGGCTCCTCAGCCGCAGCACATGCGCGGCCTCTCTCGCGCGGTGCCTTGCATCGTACCGCTGCTCGAGCATCAAAACCTCCAGCCACCCAATATTAT>1300
```

```
                *         *         *         *         *         *         *         *         *         *
1301>CGCTCGCCGGCCGCTCGCCTCCACTGCCTCCCCCCCGGTCGCTCCTCCACCTCGACCTGCAGCTCCCGACCGCCTCTTTCGCCCGATCGCAGGCCCTGCG>1400
1301>CGCTCGCCGGCCGCTCGCCTCCACTGCCTCCCCCCCGGTCGCTCCTCCACCTCGACCTGCAGCTCCCGACCGCCTCTTTCGCCCGATCGCAGGCCCTGCG>1400
1301>CGCTCGCCGGCCGCTCGCCTCCACTGCCTCCCCCCCGGTCGCTCCTCCACCTCGACCTGCAGCTCCCGACCGCCTCTTTCGCCCGATCGCAGGCCCTGCG>1400

                *         *         *         *         *         *         *         *         *         *
1401>CTGATTCGTCCCACCGCTCGCTCAATTGGGCTCCAATCGCAGCTTGGACTTGGGGCCTTTTCAGTCACGGCATCATTCAGCCCAAGGCCGGTCGAGCAGA>1500
1401>CTGATTCGTCCCACCGCTCGCTCAATTGGGCTCCAATCGCAGCTTGGACTTGGGGCCTTTTCAGTCACGGCATCATTCAGCCCAAGGCCGGTCGAGCAGA>1500
1401>CTGATTCGTCCCACCGCTCGCTCAATTGGGCTCCAATCGCAGCTTGGACTTGGGGCCTTTTCAGTCACGGCATCATTCAGCCCAAGGCCGGTCGAGCAGA>1500

                *         *         *         *         *         *         *         *         *         *
1501>GGCAGCTTCGCAAACCCCCCCGGTTCGGTTCGTTTTGCGGACGAGGCCCTTTTGAGCACTTGGAGCTTCTGCCAGTTACCTAACCGGCGGAAATATTTTC>1600
1501>GGCAGCTTCGCAAACCCCCCCGGTTCGGTTCGTTTTGCGGACGAGGCCCTTTTGAGCACTTGGAGCTTCTGCCAGTTACCTAACCGGCGGAAATATTTTC>1600
1501>GGCAGCTTCGCAAACCCCCCCGGTTCGGTTCGTTTTGCGGACGAGGCCCTTTTGAGCACTTGGAGCTTCTGCCAGTTACCTAACCGGCGGAAATATTTTC>1600

                *         *         *         *         *         *         *         *         *         *
1601>TTTCCTACTCTGCTCTCTTCCCTTGTCCCGACGGTTCTGTTCAGACGAAGTAATAGCACCAGCAACGACAGCATCTGCCCGCACTTGGTAGGCTGCGCGC>1700
1601>TTTCCTACTCTGCTCTCTTCCCTTGTCCCGACGGTTCTGTTCAGACGAAGTAATAGCACCAGCAACGACAGCATCTGCCCGCACTTGGTAGGCTGCGCGC>1700
1601>TTTCCTACTCTGCTCTCTTCCCTTGTCCCGACGGTTCTGTTCAGACGAAGTAATAGCACCAGCAACGACAGCATCTGCCCGCACTTGGTAGGCTGCGCGC>1700

                *         *         *         *         *         *         *         *         *         *
1701>TTCTGCATTGCCTTGGAGAACAGAGGTTGACAGCTGCAGGAGCCCCCAGCAGCTGACCGGAGGCAATCTCCCCCCGTACATCTCCGCCCTACTTCTCACG>1800
1701>TTCTGCATTGCCTTGGAGAACAGAGGTTGACAGCTGCAGGAGCCCCCAGCAGCTGACCGGAGGCAATCTCCCCCCGTACATCTCCGCCCTACTTCTCACG>1800
1701>TTCTGCATTGCCTTGGAGAACAGAGGTTGACAGCTGCAGGAGCCCCCAGCAGCTGACCGGAGGCAATCTCCCCCCGTACATCTCCGCCCTACTTCTCACG>1800

                *         *         *         *         *         *         *         *         *         *
1801>CCTTTCGGCCTTCTTTTGGGCAATCGAGTTTCGATAATACCTTTCTTGGGAGCTCCCATCACGGAATCATACACCTCACCTTGCCGTGCTCGGCCTCCAG>1900
1801>CCTTTCGGCCTTCTTTTGGGCAATCGAGTTTCGATAATACCTTTCTTGGGAGCTCCCATCACGGAATCATACACCTCACCTTGCCGTGCTCGGCCTCCAG>1900
1801>CCTTTCGGCCTTCTTTTGGGCAATCGAGTTTCGATAATACCTTTCTTGGGAGCTCCCATCACGGAATCATACACCTCACCTTGCCGTGCTCGGCCTCCAG>1900

                *         *         *         *         *         *         *         *         *         *
1901>CCTACGGCTAAGCATCGTCTCCTTTTGCGCTGAGTTGGTCTCGAGCCCAGCCTTGGTGCTGCCTTGGCGATAGTACCACCATCTCTCACAAGTTACTCGA>2000
1901>CCTACGGCTAAGCATCGTCTCCTTTTGCGCTGAGTTGGTCTCGAGCCCAGCCTTGGTGCTGCCTTGGCGATAGTACCACCATCTCTCACAAGTTACTCGA>2000
```

1901>CCTACGGCTAAGCATCGTCTCCTTTTGCGCTGAGTTGGTCTCGAGCCCAGCCTTGGTGCTGCCTTGGCGATAGTACCACCATCTCTCACAAGTTACTCGA>2000

* * * * * * * * * *

2001>**CCATCACAGGCGCGACCATGTCTGCGCCGTATTGGGGTTCATTGCCCCAGCCCAAGGGCATCCACAGCCGGCGAGTCTCAGCAGATTACACTAATGAA**>2098
2001>CCATCACAGGCGCGACCATGTCTGCGCCGTATTGGGGTTCATTGCCCCAGCCCAAGGGCATCCACAGCCGGCGAGTCTCAGCAGATTACACTAATGAA>2098
2001>CCATCACAGGCGCGACCATGTCTGCGCCGTATTGGGGTTCATTGCCCCAGCCCAAGGGCATCCACAGCCGGCGAGTCTCAGCAGATTACACTAATGAA>2098

* * * * * * * * * *
